# How far are the new wave of mRNA drugs from us? mRNA product current perspective and future development

**DOI:** 10.3389/fimmu.2022.974433

**Published:** 2022-09-12

**Authors:** Qiongyu Duan, Tianyu Hu, Qiuxia Zhu, Xueying Jin, Feng Chi, Xiaodong Chen

**Affiliations:** Department of Oncology, Shengjing Hospital of China Medical University, Shenyang, China

**Keywords:** mRNA, drug, targeted therapy, development, preclinical studies

## Abstract

mRNA products are therapies that are regulated from the post-transcriptional, pre-translational stage of a gene and act upstream of protein synthesis. Compared with traditional small molecule drugs and antibody drugs, mRNA drugs had the advantages of simple design, short development cycle, strong target specificity, wide therapeutic field, and long-lasting effect. mRNA drugs were now widely used in the treatment of genetic diseases, tumors, and viral infections, and are expected to become the third major class of drugs after small molecule drugs and antibody drugs. The delivery system technology was the key to ensuring the efficacy and safety of mRNA drugs, which plays an important role in protecting RNA structure, enhancing targeting ability, reducing the dose of drug delivery, and reducing toxic side effects. Lipid nanoparticles (LNP) were the most common delivery system for mRNA drugs. In recent years, mRNA drugs have seen rapid development, with the number of drugs on the market increasing each year. The success of commercializing mRNA vaccines has driven a wave of nucleic acid drug development. mRNA drugs were clinically used in genetic diseases, oncology, and infectious diseases worldwide, while domestic mRNA clinical development was focused on COVID-19 vaccines, with more scope for future indication expansion.

## Introduction

### Definition and classification of mRNA products

**Figure 1:**
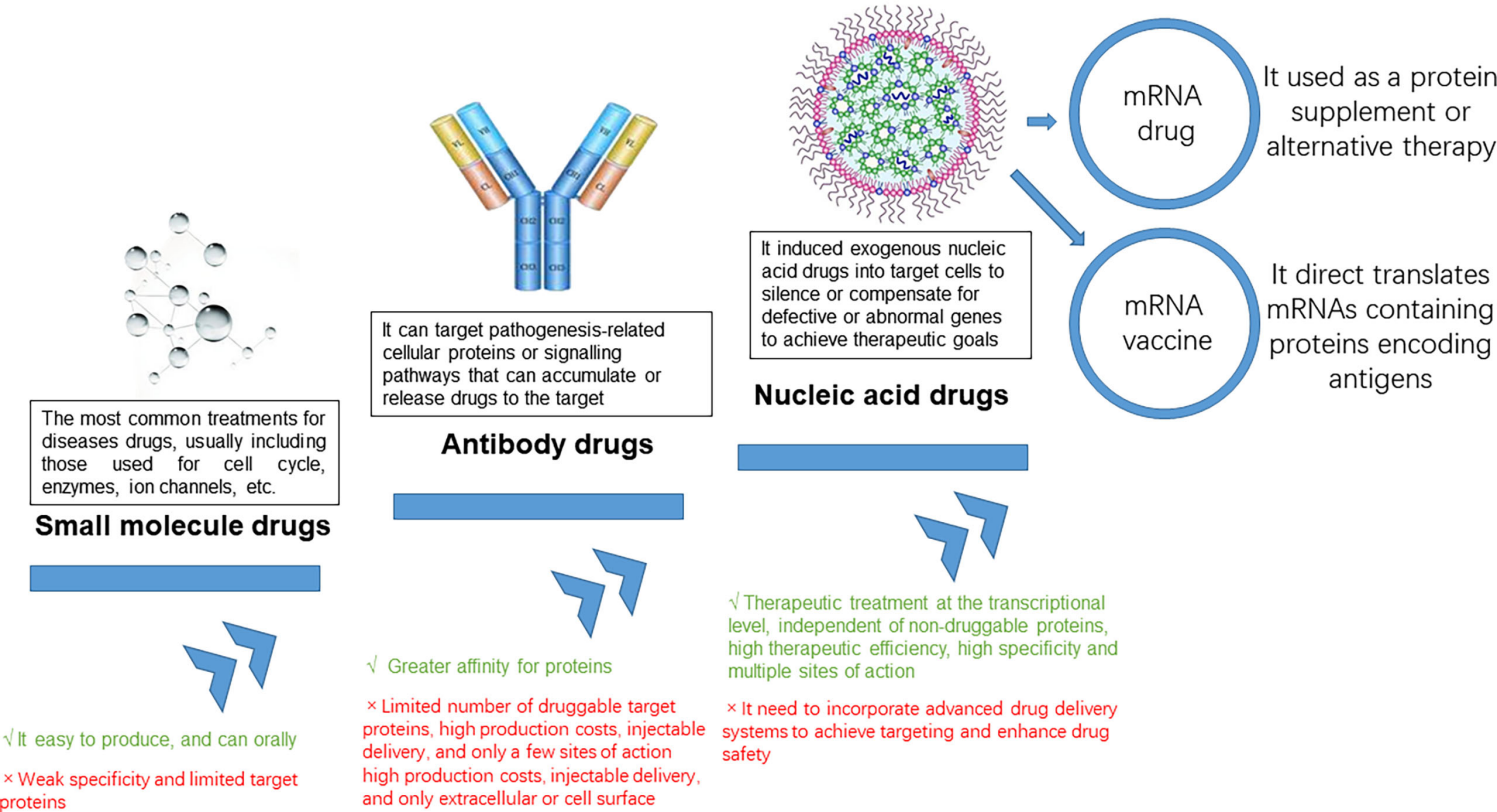
Trends in the evolution of therapeutic drugs.

Nucleic acids were the carriers of genetic information for all living organisms and include two major categories: deoxyribonucleic acid (DNA) and ribonucleic acid (RNA) (1). With the development of molecular biology, it has been discovered that in addition to protein-coding nucleic acid sequences, there were also a large number of non-coding sequences that play an important regulatory role in human life activities, such as promoters, enhancers, nucleases, miRNAs, etc (2–6). mRNA products can be divided into mRNA vaccines and mRNA drugs (7, 8) (**Figure 1**). Drugs that utilized the translational or regulatory functions of mRNA molecules as interventions for diseases are mRNA drugs (9). Therefore, compared to traditional small molecule drugs and antibody drugs, mRNA drugs could intervene at the source by inhibiting the expression of disease-related genes as pathological proteins or introducing genes that can express normal proteins to compensate for the lack of functional proteins, which had the characteristic of “treating the symptoms and the root” (10). In addition, mRNA drugs had the advantages of high therapeutic efficiency, low drug toxicity, and high specificity, and currently had great potential in the treatment of metabolic diseases, genetic diseases, cancer, and prevention of infectious diseases (11).

### Principle of mRNA drug therapy

**Figure 2:**
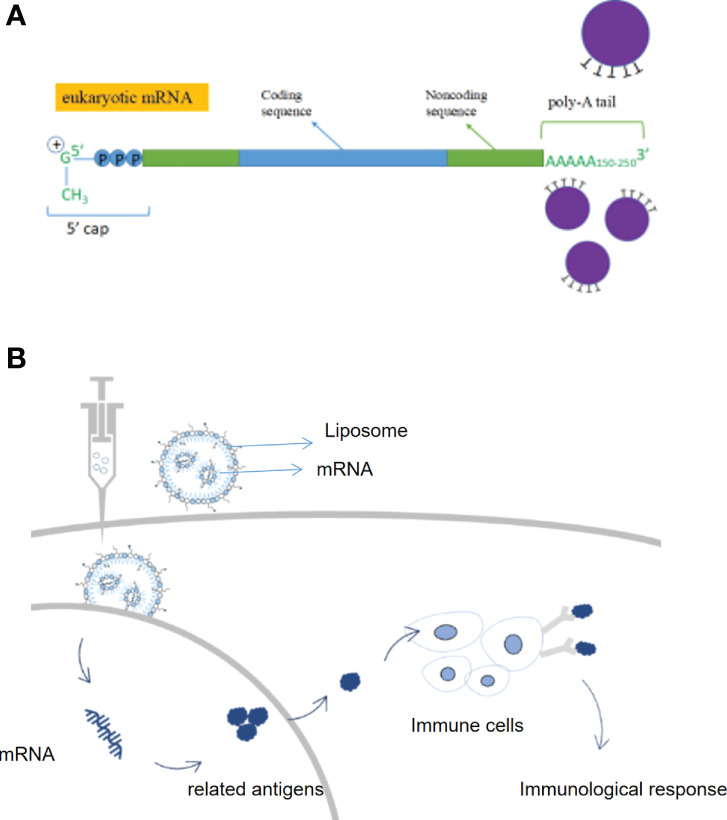
Structure and therapeutic principles of mRNA drugs. (A) Key structures of mRNA products, (B) Mechanism of action of mRNA vaccines.

Messenger RNA (mRNA) conveyed genetic information in DNA and synthesizes proteins through translation. mRNA entered the body which was expressed by autologous cells to produce specific proteins, avoiding the influence of *in vitro* factors; it can regulate the body’s immune system through the endogenous expression of functional proteins and eliminate autologous threats, including cancer cells (12). Compared to conventional therapies, mRNA therapies have a variety of advantages such as being more targeted, simpler to synthesize, more widely adaptable, and alternative to protein therapy (13). An mRNA vaccine encoding an antigen sequence is introduced into cells *via* a delivery platform such as a lipid nanocarrier and is then translated by human cells to produce the antigen and activate an immune response (14). By endogenously expressing antigenic proteins, mRNA vaccines can induce a more widespread and effective cellular and humoral immune response, resulting in higher protection rates than conventional vaccines (15). Germinal centers are the key to a long-lasting immune response, the place where immune memory is formed, and the longer they are present, the stronger and more durable the immunity will be. Researchers point out that the success of a vaccine depends on the ability to trigger a durable, high-affinity antibody response (16) (**Figures 2A, B** **)**.

Not only does mRNA work as a vaccine, but mRNA drug replacement therapy is also one of the main mRNA therapeutic modalities (17). By synthesizing mRNA sequences *in vitro* and then delivering them into cells by delivery systems, mRNA injected into patients can initiate the production of drugs in the patient’s cells, compensate for defective genes/proteins, transform various protein drugs such as monoclonal antibodies, enzymes, and cytokines in the form of mRNA platforms and be used to treat a wide range of diseases such as metabolic disorders, heart disease, and immuno-oncology (1, 12, 13). Compared to recombinant proteins or small molecules, RNA therapeutics are relatively simple to develop and manufacture and are more cost-effective. In addition, RNA sequences can be easily modified to personalize RNA therapies. mRNA can also be applied in the field of gene editing. mRNA is capable of encoding nucleases like Cas9 in the cytoplasm, with the advantage of rapid expression and no risk of gene integration (5). Cell therapy is one of the main therapeutic applications of mRNA (18). After obtaining cells from a cell bank, they are therapeutically modified *in vitro* with mRNA encoding the desired protein, and the mRNA-enhanced cells are then reinfused into the patient to treat the disease. Several cell therapies that use mRNA are currently in clinical trials. Examples are TriMix-based immunotherapy (ECI-006), autologous cell therapy CAR-T MCY-M11 (MaxCyte), and Cartesian therapy (19). mRNA has the potential to prevent and treat a wide range of diseases, and as a breakthrough technology platform, is expected to partially replace traditional drugs and vaccines, opening up new therapeutic frontiers and revolutionizing new treatments (20).

### Prospects for mRNA therapeutics

In the field of vaccines against infectious diseases, mRNA vaccines have excellent prophylactic potential, can rapidly modify sequences to respond to the emergence of new mutant strains, are effective against immune escape pathogens, and do not integrate into the genome, offering safety advantages (21). In the field of therapeutic cancer vaccines and immuno-oncology vaccines, mRNA vaccines can activate the host’s anti-tumor immunity and also modulate the immunosuppressive tumor microenvironment in solid tumors, thereby inhibiting tumor growth and promising to prolong clinical survival and reduce cancer recurrence rates (22, 23). In the field of protein replacement therapies, the mRNA platform is suitable for most protein-based drugs. It can avoid unnecessary immune responses, has a broader therapeutic scope, and is less difficult to develop than protein-based therapies (24).

mRNA was discovered in 1961, and mRNA received little attention at the time due to its low stability (25). As mRNA has become better understood, it has now become an ideal platform for the treatment of many disease areas (26). mRNA therapy has unique advantages over traditional therapies. Because mRNA is translated into target proteins within the cell, the mRNA-encoded proteins can target both intracellular and cellular membranes, making them more effective than injected proteins, which can only work through extracellular interactions (27). The properties of mRNA make it free from potential infection, insertional mutations, and genomic integration risks. mRNA can be degraded by normal cellular processes, reducing the risk of toxicity. mRNA’s immunogenicity can be down-regulated to further improve safety (28). mRNA vaccines have the potential for rapid and scalable production due to the high yield of the *in vitro* transcriptional response. The approval and commercialization of mRNA vaccines during the COVID-19 epidemic has increased the scale of global mRNA production and further reduced raw material prices (29). mRNA can be used to prevent and treat different diseases and once this system has been established and validated, only the sequence of the mRNA needs to be changed for use in other products (30). This ensures that development cycles can be significantly shortened and costs can be reduced (31).

### History of mRNA therapy development

**Figure 3:**
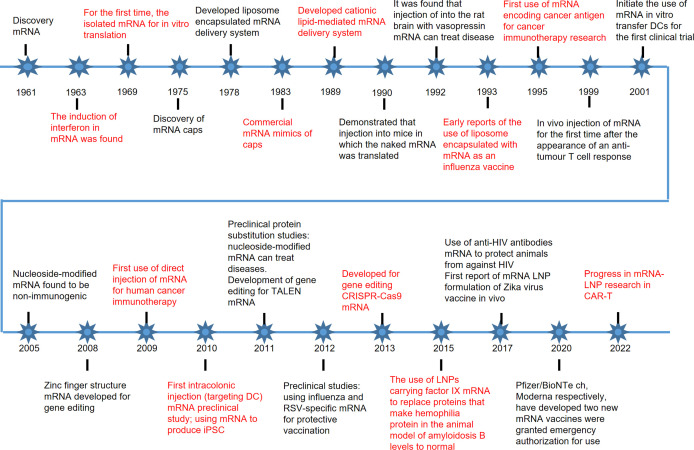
History of mRNA therapy.

Since the discovery of mRNA in 1961, the therapeutic potential of mRNA for the disease has been continuously explored (32). In 1987, Robert Malone conducted a landmark experiment in which he mixed mRNA with fat droplets and discovered that this mRNA could be taken up by frog embryo cells, a discovery that laid the foundation for the idea that RNA could be considered a drug delivered into the body to express (33). Since the 1970s, delivery systems have been optimized and updated, and there has been increasing interest in the immune response to mRNA once it enters the body (34). It was found that nucleoside modifications could reduce the immunogenicity of mRNAs and allow them to be accepted by the body for the treatment of disease (35). As the ability of mRNA to treat disease is explored, different mRNA therapies are emerging, including alternative therapies, mRNA vaccines, immunotherapies, gene editing therapies, etc (36–38). Pre-clinical human trials have been conducted for different therapies, and disease-specific mRNA vaccines and proven delivery systems have been developed, which may be developed on a large scale for disease prevention and treatment in the future (**Figure 3**). mRNA therapies break through many of the limitations of traditional therapies and have the potential to become a novel therapeutic alternative to traditional treatments (39, 40).

## Current mRNA drug development

Now, there are two mRNA products on the market globally(**Table 1**). In December 2020, the FDA approved two new mRNA COVID-19 vaccines from Pfizer with BioNTech and Moderna for emergency use, with formal approval for both products in 2021 and 2022, respectively (41, 42).

**Table 1:** Global marketed mRNA products.

Categorie	Common name	Trade name	Company	Target	Delivery system	Year of approval
mRNA	Tozinameran	Comirnaty	Prizer@BioNtech	SARS-CoV-2	LNP	2020
mRNA	Elasomeran	Spikevax	Moderna	SARS-CoV-2	LNP	2020

The mRNA drugs currently under development can be divided into three main categories by use and drug type: prophylactic vaccines, therapeutic vaccines, and therapeutic drugs (43–45). According to statistics, there are 56 mRNA drugs in the clinical pipeline worldwide, with R&D mainly focused on vaccines, accounting for about 84%, while therapeutic drugs account for about 16%. Except for the mRNA COVID-19 vaccine, which is urgently marketed, most others are in the early stages. mRNA R&D is mainly in clinical phase I, accounting for about 40% of the total number (46).

The range of applications for mRNA is extremely broad. The main applications for mRNA currently include three major directions: immunotherapy, protein replacement therapy, and regenerative medicine therapy (47–49). Among the immunotherapies, tumor immunotherapy and infectious vaccines are the most popular and mature, and the mRNA pipeline under development mainly targets genetic diseases such as infectious diseases, oncological diseases, and rare diseases. For delivery systems, the commonly used carriers for mRNA include viral-based delivery systems, fisetin, and lipid nanoparticles, among which lipid nanoparticle delivery systems are the most commonly used carriers, accounting for about 81% of the total (50). mRNA drug development’s main technical threshold lies in stability and delivery, and the delivery system is crucial to the effect of mRNA. The rapid responsiveness, universal adaptability, and rapid production capacity of mRNA-based drugs make them highly promising for use in a wide range of diseases (51).

Since the outbreak of the COVID-19 epidemic, domestic listed pharmaceutical companies have started a boom in collaborating with mRNA companies to develop vaccines. In terms of COVID-19 vaccines, domestic Watson/Aibo/ARCoV from the Academy of Military Sciences is in clinical phase III (52), BNT162b2 from Fosun Pharma and BioNTech is in clinical phase II (53). The domestic vaccine industry is expected to compete with overseas markets at the same stage; most of the other indications are in the pre-clinical/early stage. At present, the domestic mRNA research field is dominated by COVID-19 vaccines, while global mRNA drug indications are distributed in genetic diseases, COVID-19, and other infectious diseases, compared to the future indications of domestic mRNA drugs, there is more room for expansion (54).

**Figure 4:**
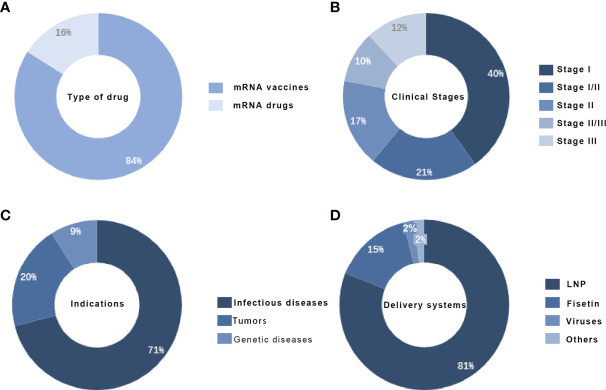
Global clinical mRNA distribution, (A) Global mRNA product type distribution (B) Global mRNA drug clinical staging distribution (C) Global mRNA drug indication distribution(D) Global mRNA drug delivery system distribution.

The year 2020 was a breakthrough year for mRNA, with two mRNA COVID-19 vaccines developed by Pfizer BioNTech and Moderna, respectively, receiving emergency use authorizations. mRNA COVID-19 vaccines will generate global sales of US$58.7 billion in 2021, and both far exceed sales of other COVID-19 vaccines (55, 56). mRNA technology can be used primarily in vaccines, immunotherapies, and protein replacement therapies. In the case of COVID-19 vaccines, mRNA vaccines offer advantages in terms of development efficiency and protection rates. In the short term, the market for preventive vaccines will be dominated by COVID-19 products, while in the medium to long term, mRNA vaccines will expand into the prevention of more infectious diseases, such as influenza virus vaccines, influenza respiratory syncytial virus vaccines, and malaria vaccines, due to their advantages in terms of the number of targets, efficacy, safety and manufacturing processes (57, 58). In addition to preventive vaccines, immunotherapy for tumors is also a hot topic of research in mRNA technology (48). Traditional immunotherapy requires the injection of antigens into the body, which is difficult and expensive to synthesize. In the field of oncology treatment, there is a large number of patients and unmet clinical needs, and the market size for therapeutic oncology vaccines will continue to climb in the future (**Figure 4**). Overall, with improvements and advances in delivery technology and stability, mRNA has a very strong competitive advantage and has the potential to become a cross-application product (59).

## mRNA drug key development technologies and challenges

### Key issues and challenges in mRNA product development

According to the central law, mRNA theoretically had the potential to be expressed as any kind of protein through the ribosome and therefore can be used to treat a wide range of diseases, and mRNA is cheaper to produce than recombinant protein drugs (60). However, mRNA technology still needs to address the pain points of immunogenicity, efficiency in expressing proteins in vivo, and ultimately scale up production, so innovations in key development technologies such as sequence design, synthetic design of LNP delivery systems, and optimization of manufacturing processes could facilitate the development of mRNA drugs (61). mRNA design sequence optimization - both coding and non-coding sequences can be optimized (62). The translation efficiency of mRNAs can be improved by optimizing the coding sequence (63). Optimizing non-coding sequences can also increase translation efficiency and mRNA stability(2). However, the process of sequence optimization requires specific skills and experience. The immune system can recognize unmodified single-stranded RNA, causing a decrease in protein expression and the development of reactogenicity (64). Translation efficiency can be improved by introducing modified nucleotides, the most commonly used modified nucleoside, N1-methyl-pseudouridine, requires a patent license (65). As mRNA is a negatively charged biomolecule, it is difficult to pass through the negatively charged lipid bilayer on the surface of cell membranes (66). In addition, mRNA is susceptible to phagocytosis by immune system cells and degradation by nucleases, as well as having intracellular release challenges. Therefore, an efficient and safe delivery system is essential for mRNA drugs. LNP is currently the most clinically advanced mRNA delivery vehicle and is highly feasible and patentable (67). However, its synthetic design needs to be optimized to address its toxicity and tendency to aggregate and leak. The purity of mRNA has a significant impact on efficacy and safety, and efficient purification methods need to be developed to improve mRNA purity and remove impurities such as double-stranded RNA, truncated mRNA, and DNA residues (68). mRNA particle size uniformity can improve the stability of the LNP delivery system (69). In large-scale production, specific encapsulation techniques can improve encapsulation efficiency, ensure consistent quality and save production costs (70).

### mRNA development technologies

The core technical challenges at the design and synthesis level are mastering platform-based computational capabilities, and 5’ end-capping and UTR region nucleotide modifications are key points (71). At the modification level, chemical modifications improve drug stability and reduce toxicity (72). At the level of delivery system synthesis and design, the synthesis of lipid nanoparticles mRNA-LNP is a key focus of R&D in the mRNA field (20). mRNA vaccine or drug scale-up production is highly reproducible, with successful sequence modification and delivery system assembly at the core. The upstream and downstream chains of scale-up production are also particularly important, involving hundreds of enzymes, nucleotides, liposomes, and other raw materials, for example, and presenting many technical challenges such as the difficulty of mass production of raw materials and high barriers to production equipment (**Table 2**).

**Table 2:** Clinical pipeline of China mRNA drugs in development.

Drug Name	Indications	Clinical Phase	Year
COVID-19 mRNA vaccine ARCoV	COVID-19	Phase IIIb	2021
COVID-19 mRNA vaccine	COVID-19	Phase II	2022
COVID-19 mRNA vaccine (LVRNA009)	COVID-19	Phase II	2021
SARS-CoV-2 mRNA vaccine (BNT162b2)	COVID-19	Phase II	2020
Personalized tumor vaccine with mRNA encoding a nascent antigen/Tremelimumab injection	Advanced non-small cell lung cancer	Phase I	2021
COVID-19 mRNA vaccine SW0123	COVID-19	Phase I	2021
Personalized tumor vaccines with mRNA encoding nascent antigens	Advanced malignant solid tumors	Phase I	2019

For the new mRNA COVID-19 vaccines already licensed, accessibility was further limited by refrigeration conditions. If billions of people are to be vaccinated globally, a vaccine with better heat resistance is required. In preclinical studies, CureVac has demonstrated that the sequence-optimized rabies virus vaccine in development, RABV-G, could remain stable for several months in the region between -80°C and 70°C (73). Furthermore, two investigational mRNA COVID-19 vaccines had now been reported to be able to remain stable in a room temperature environment (74, 75). If these heat-resistant vaccine candidates achieve positive results in clinical trials, it is expected to improve the global accessibility of mRNA vaccines (**Table 3**).

**Table 3:** Core technical challenges in the development of mRNA drugs.

	Definition	Core technical challenges	Purpose
Design synthesis	Sequencing analysis for target indications, design synthesis, and screening of corresponding mRNA sequences	• Key steps in biosynthesis and development of key tool enzymes (capping, chemical modifications, etc.) •Exploration of chemical synthesis and integration with biosynthesis •Development of proprietary mRNA sequence design solutions to enhance expression and druggability	•Enhanced target binding specificity •Reduces off-target toxicity
Chemical modifications	A technique for structurally optimizing mRNA drugs using chemical methods to modify and optimize their properties *in vivo*.	•Phosphate modification •Glycosyl modifications • Base modifications	•Improving stability •Reduces non-specific toxicity
Delivery systems	Technology to enhance drug concentration and bioavailability in target tissues by using vectors to encapsulate and transport mRNA to its site of action in a highly efficient manner with low toxicity	• Barely modified RNA delivery systems, nanoliposome delivery systems, polymer delivery systems, targeted molecular conjugate linkage delivery systems, peptide and exosome delivery systems, etc. • Optimised synthesis design to address the delivery system’s toxicity and tendency to aggregate and leak	• Protecting RNA stability • Improving drug targeting and reducing off-target toxicity • Enhances tissue and cell penetration •Reduced drug dose •Enables flexible drug delivery •Reducing delivery system-mediated drug toxicity
Amplify production	Design plasmids, amplify cultures, harvest and purify mRNA and encapsulate vectors to meet production needs using relevant technologies and process strategies	• Difficulty in mass production of cap structure analogs, modified nucleotides, and ionizable lipids in raw materials • High barriers to microfluidic equipment for mRNA-LNP synthesis in production facilities	•Ensuring efficiency and scalability •Maximising yield, recovery, and impurity removal •Enabling mRNA drug product development and commercialization

### Technical analysis of mRNA drug delivery systems

**Figure 5:**
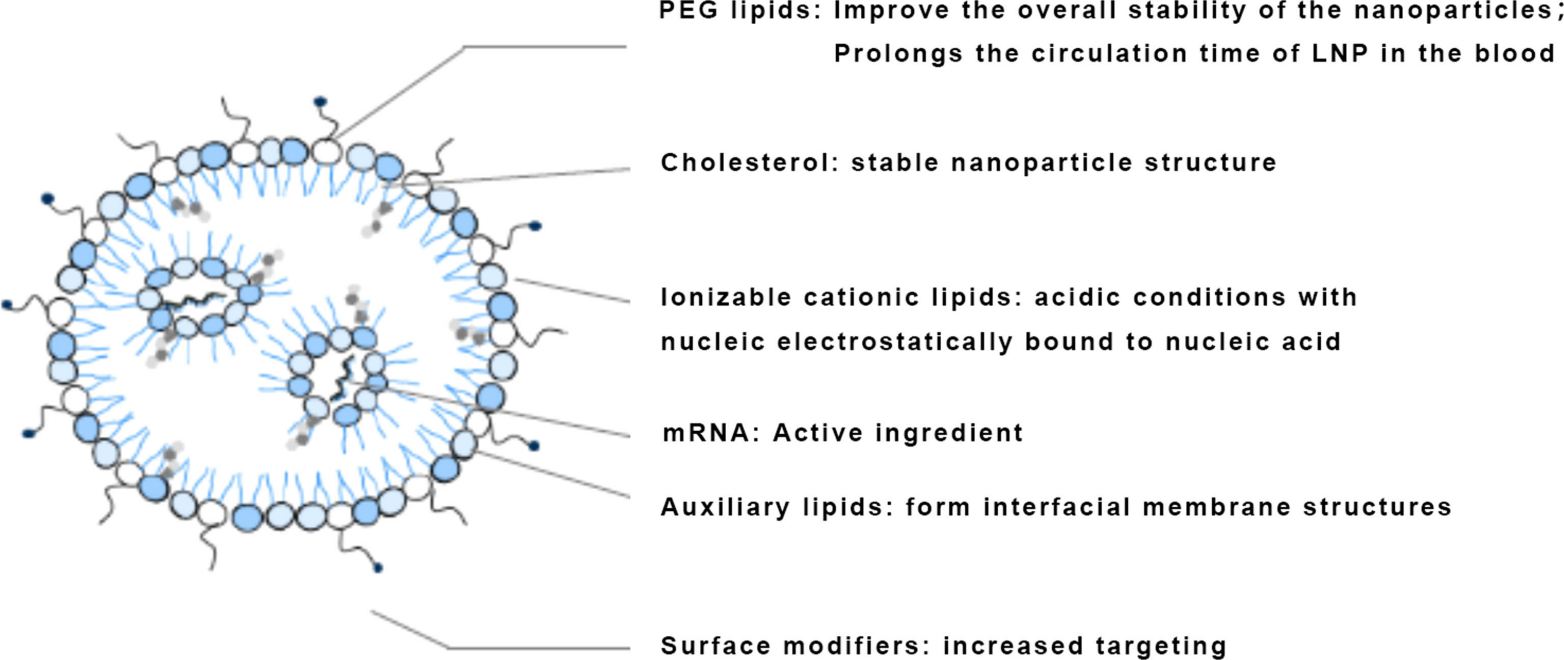
Diagram of the LNP delivery system.

The development of mRNA delivery systems has gone through the stage of fisetin, a naturally occurring cationic protein that can complex negatively charged mRNA molecules into nano-sized nucleic acid particles to protect mRNA from degradation by RNA enzymes in serum, which was discovered in 1961 (32). Cationic polymers neutralize the negative charge of the nucleic acid drug to increase the efficiency of entry into the cell (76). Polyethyleneimine (PEI) was a common polymer used for gene delivery, but the high molecular weight of PEI resulted in high cytotoxicity, making its successful use for vaccines in a limited number of animal studies, and chemical modifications of PEI were continually being explored (77). Liposomes began to be used as a carrier-optimized route of administration in 1978, and cationic lipids were first proposed to mediate mRNA transcription in 1989, until the successful launch of mRNA COVID-19 vaccines in 2020 (78–80). Lipids are currently the most widely used class of delivery systems for nucleic acid drugs, including liposomes and lipid nanoparticles, the most commonly used being LNPs containing ionizable lipids (**Figure 5**). The first generation was 1,2-dioleylidene-3-dimethylaminopropane (DLinDMA) and the second generation included DLin-MC3-DMA, which was further optimized based on the first generation and led to the development of drugs such as Patisiran and ALN-PCS (81). In contrast to the first generation, DLin-MC3-DMA has a unique pH-dependent charge-variable property: it is positively charged under acidic conditions and electrically neutral under physiological pH conditions (82). It protonates in the acidic environment of endosomes or lysosomes, promoting membrane fusion of LNP with endosomes and allowing mRNA to escape from endosomes/lysosomes to function (51) (**Table 4**).

**Table 4:** China and international mRNA-LNP products.

Company	Technical sources	Drug indications	Product	Progress
BioTech	Genevant Authorisation	COVID-19	BNT162b2 (Tozinameran)	Approved in market
Moderna	Acuitas Authorisation	COVID-19	mRNA-1273 (Elasomeran)	Approved in market
BioTech	Genevant Authorisation	COVID-19	BNT162b1	Phase II/III
ABOGEN	Self-developed	COVID-19	ARCoV	Phase IIIb
Moderna	Acuitas Authorisation	COVID-19	mRNA-1647	Phase III
Moderna	Acuitas Authorisation	Zika Virus	mRNA-1893	Phase II
Argorna	Self-developed	COVID-19	RBMRNA-176	Phase II

The advantages of using an LNP delivery system include the following: the use of LNP removes the risk of infection, oncogenicity, and immunogenicity compared to viral vectors (83); LNP can be introduced into the target cell by decorating the cell surface with ligands; LNP prevents mRNA degradation and aids endocytosis and endosomal escape, thus enhancing antigen expression and the efficiency of mRNA vaccination; adjuvants can be added to LNP to aid immune activation and potential immune response (20).

### The side effects of mRNA vaccines

Regarding mRNA side effects, there had been reported high rates of vaccination-related side effects, including pericarditis, myocarditis, neurological inflammation, and autoimmune hepatitis (84–87). Although published data from clinical study sponsors and others suggested that these side effects are not related to the vaccine itself (88). mRNA vaccine side effects occur at a rate of 80-90%, with moderate to severe side effects reaching around 10%, although the majority are common low-grade side effects (89). Studies from academic institutions showed that the majority of side effects are due to LNP components such as PEG and ionized lipids, with both natural and adaptive immunity involved. To improve vaccine efficacy and limit side effects, researchers were changing the four components that make up the lipid nanoparticles. Each particle contains ionizable lipids that bind messenger ribonucleic acid (mRNA), and once inside the body, their charge shifts from positive to neutral to limit the toxicity of the particles. The other three lipids contribute to their structure and stability. Auxiliary lipids also help the particles fuse with the cell, cholesterol helps them escape the cell’s endosome, and polyethylene glycol (PEG) liposomes prevent them from aggregating, thus prolonging their action. Dan Peer has also developed novel ionized lipid libraries with atypical structures (90, 91). In unpublished experiments, they appear to enable better mRNA vaccines with fewer side effects and prolong their stability at room temperature.

Other improvements might come from facilitating the uptake of LNPs into the cell and then enhancing their ability to break free of the cell membrane vesicles, known as endosomes, that carried them into the cell (92). The vast majority of LNPs are trapped in these vessels and then destroyed or expelled without delivery of the vaccine payload, meaning that a significant amount of RNA is not used. The shape of ionizable lipids affects the ability of LNPs to destroy endosomes, and cholesterol is another lipid in LNPs (93). Sahay reports that the use of different forms of cholesterol can improve the escape rate of LNPs, and Sanofi has begun to evaluate its bespoke LNPs positively in human trials (94). which announced that a lipid formulation proved to be more effective in initiating anti-flu immunity. Other companies, including BioNTech and Arcturus Therapeutics, had begun exploring the elimination of polyethylene glycol, a compound that helps stabilize LNPs but is also associated with certain types of adverse vaccine reactions (95). At the same time, more and more companies were focusing on optimizing lipids to deliver mRNA to treat disease, rather than prevent it. This requires access to mRNAs that can encode disease-correcting proteins into the precise cells and tissues, not just the liver, as current preparations of LNPs tend to end up after infusion. Delivery of LNPs will be key to truly expanding the reach of mRNA beyond prophylactic vaccines.

## mRNA drug industry chain and development trend

### The mRNA drug industry chain

**Figure 6:**
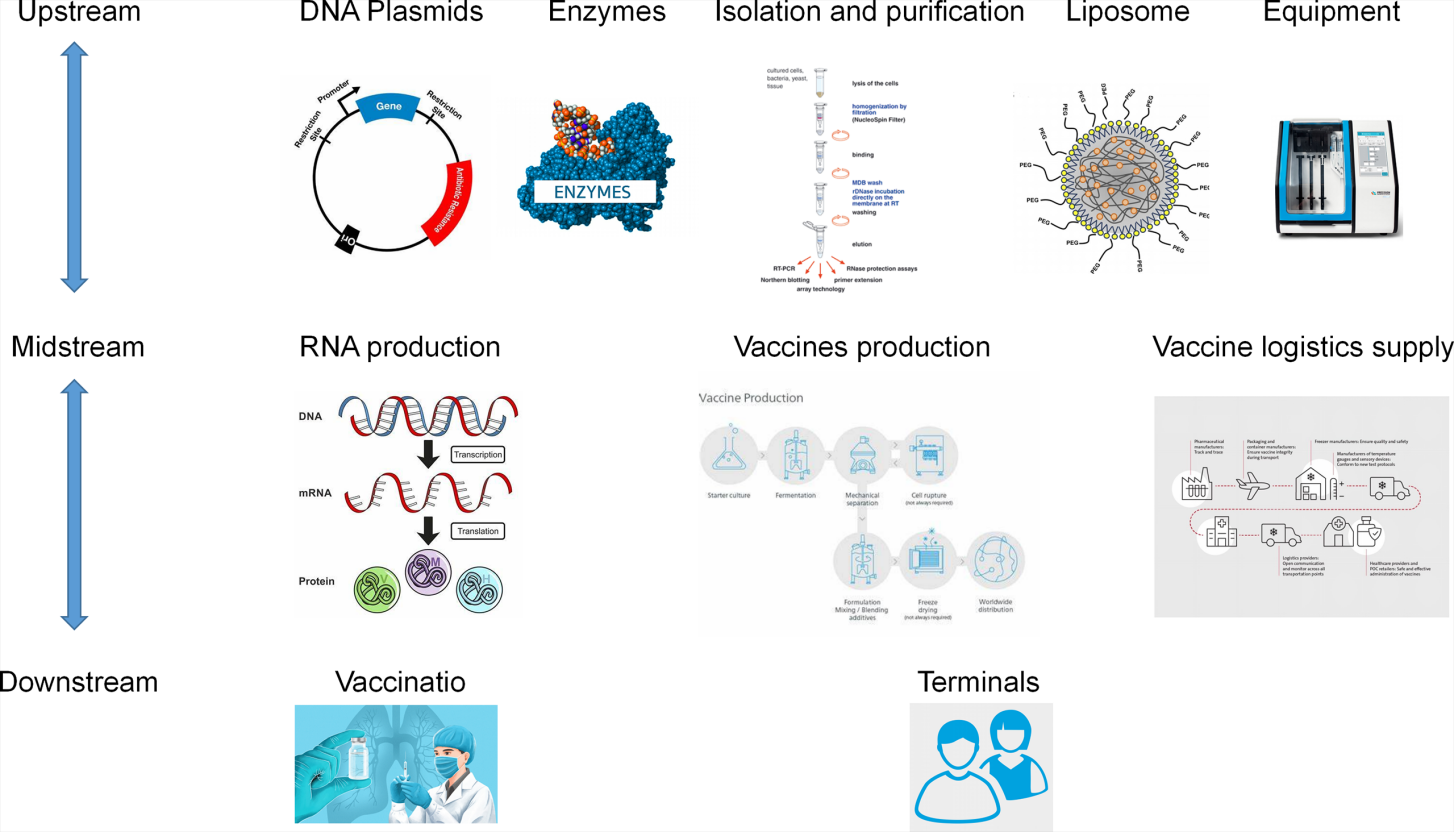
mRNA drugs industry chain.

The mRNA drug industry chain covers the upstream preparation of raw materials, the midstream development and production of biopharmaceutical companies, and the downstream commercialization of products for patients(**Figure 6**). mRNA technology faces challenges in sequence optimization and synthesis, delivery systems, and large-scale formulation and storage (96). Continuous optimization of technology will continue to drive the rapid expansion of the mRNA market. In terms of mRNA synthesis and sequence optimization, sequence optimization is required to improve the stability and expression efficiency of mRNA due to its relatively short half-life, and further sequence improvement is required to control immunogenicity as mRNA can induce intrinsic immunogenicity (97). mRNA optimization directions include: 5’ cap addition, 5’ untranslated region (5’UTR), open reading frame (ORF) region, 3’ untranslated region (3’UTR) and polyA tail structure (72). In terms of delivery systems, liposomal nanoparticles are currently the preferred mRNA delivery system, which is virtually non-toxic to most cells, but can still have side effects on the liver in some cases, and future delivery systems need to be further optimized (98). In terms of large-scale preparation and storage, although the mRNA production process is not complex, it is relatively early in the field and a stable and controlled supply chain for large-scale production has not yet been established. mRNA products require ultra-low temperature transport and storage, breakthroughs in storage methods will facilitate their rapid development.

To date, clinical studies had demonstrated that siRNA drugs can be delivered effectively *in vivo* using LNPs, and with the approval of the LNP-mediated siRNA therapeutic agent Onpattro® (patisiran), the use of LNPs for delivery was one of the first ideas in the field of mRNA delivery (99). Two LNPs, mRNA-1273 and BNT162b2, had now been used in clinical trials. In recent years, LNPs had also shown great potential for use in self-amplifying mRNA vaccines and conventional non-replicating mRNAs. mRNA-LNP complexes delivered by LNPs systems were primarily targeted at the liver, however, the mechanism of mRNA escaping into the cytoplasm was not fully understood. New directions for the continued development of LNPs systems were focused on ionizable lipids and formulations, all of which combinations of optimization areas are almost limitless, and success in any of these parameters could beneficial effects and provide new approaches to successful vaccine disease prevention (76).

However, there were still problems to be overcome in the delivery of mRNA vaccines, such as the toxicity of some lipid formulations and the difficulty of reaching immune cells in specialized secondary lymphoid organs. Multi-antigen vaccines were also a major challenge in mRNA vaccine development, for example, the number of LNP:mRNA ratios are typically around 10:1 to 30:1, and multi-antigen candidates require large numbers of LNPs, which have inherent adjuvant properties, so safety and tolerability may limit the development of multi-antigen mRNA vaccines (100).

With the continuous maturation of mRNA *in vitro* synthesis and delivery technology in recent years, the stability and translation efficiency of mRNA drugs have improved significantly, and mRNA technology has been able to develop rapidly (101). Also driven by the emergence of a large number of biotechnology companies and a boom in capital market investment and financing, the future mRNA drug market will show the following trends: Expansion of therapeutic areas, mRNA is a revolutionary platform for the production of antigens or drugs using cells and is suitable for most vaccine products. There are proven mRNA vaccine platforms that manufacturers can apply to other diseases by simply changing the mRNA sequence (102). As a result, mRNA-based therapies can be rapidly scaled up to a wide range of other diseases. mRNA therapies are also potential alternatives to many future protein-based drugs such as cytokines and even antibodies, and mRNA-based therapies have the potential to be further extended to many other disease treatments (103). In addition, mRNA drug development could also target some of the targets that are now difficult to drug, further expanding the therapeutic field. The rapid development of the upstream and downstream of the industry chain: The outbreak of the new COVID-19 epidemic in 2020 has accelerated the popularity and commercialization of mRNA technology, with countries increasing their investment in mRNA technology and giving rise to the birth of mRNA biotechnology companies, promoting the construction and improvement of mRNA and drug technology platforms, while bringing about an explosion of opportunities upstream and downstream of the mRNA industry chain, driving the development of mRNA drugs, including upstream raw materials, midstream research and development and manufacturing, and downstream drug discovery (104). R&D and manufacturing, as well as downstream distribution and transportation. Improved efficacy and safety: Compared with traditional vaccines, mRNA vaccines have higher efficacy but also have relatively greater side effects. mRNA drug efficacy and safety control are also critical to clinical practice. As mRNA research progresses and new technologies such as AIs are developed, better mRNA codon optimization, the use of modified bases, and improvements in delivery systems can produce higher immune responses at lower doses. The use of new mRNA platform technologies, such as self-amplifying mRNA, is also a potential approach to reduce the dose and ensure clinical effectiveness while reducing side effects (105). Gradual completion of R&D system: Currently, mRNA pharmaceutical companies have formed complete mRNA vaccine teams, optimized mRNA high-throughput synthesis platforms, improved patent systems, enriched mRNA pipelines, and advanced processes, developed leading mRNA delivery technologies, and gradually scaled up to mass production technologies (106). In the future, the mRNA industry will make breakthroughs in drug development technology and gradually improve in all aspects such as product lines, formulation technology, patent layout, and production processes (107). Cooperation in commercialization, after the start of the epidemic, many COVID-19 vaccine companies in various countries responded quickly and started rapid research and development of COVID-19 vaccines, actively introducing leading products and reaching cooperation with relevant companies to obtain commercialization rights of mRNA vaccines (108). At the same time, the different experiences and core competencies of national companies in mRNA chemical modification, targeted nucleic acid drug delivery formulation, vaccine registration, clinical research, industrialization, and marketing have led to multiple collaborations between companies.

There was a growing interest in developing combination vaccines to protect against more than one disease using a single vaccine. Non-replicating mRNAs encoded only the target antigen, while self-amplifying mRNAs vaccines also encoded the replication mechanism of the virus. The combination of CAR T-cell therapy and mRNA vaccine technology avoids some of the potential problems associated with CAR T-cell therapy in the treatment of hematological malignancies. These include the use of viral vectors that alter cellular DNA (109); T cells need to be harvested from the patient, cultured, and then reintroduced into the patient; the number of T cells that had not been genetically engineered must be reduced by chemotherapy (110). These combination therapies also extend the potential of mRNA technology beyond vaccines. Lin Jinzhong’s group at Fudan University has developed a new crown mRNA vaccine, RQ3013, capable of broadly neutralizing mutant strains of VOCs, encoding a Spike protein carrying the Alpha/Beta/Omicron Spike mutation. In mouse and NHP rhesus monkey models, it strongly triggers high titers of seronegative antibodies targeting the original strains, Alpha (B.1.1.7), Beta (B.1.351), Delta (B.1.627.2), and Omicron (B.1.529). The systematic evaluation of the immunogenicity, immunoprotective efficiency, and safety of RQ3013 in animal models confirmed that it was a promising broad-spectrum vaccine capable of targeting new crown VOCs (111).

In summary, the stability and delivery efficiency of mRNA drugs has been a constraint to drug success. As mRNA is by nature a transient molecule with a short half-life, it is easily degraded, and its large molecular weight and high negative charge density affect its permeability through cell membranes. The specific delivery of mRNA drugs to cells is therefore one of the key bottlenecks in the role of mRNA technology. As the core of mRNA drugs is specificity and stable delivery. The ideal delivery vehicle must be free from enzymatic degradation, be specifically taken up by the target cell, and finally be released from the endosome in time after entry into the cytoplasm. This ensures the stability and translation efficiency of mRNA drugs but is also a hurdle that must be overcome for mRNA drugs to reach the clinical application. This has been achieved by optimizing mRNA structural chemistry and delivery systems. To improve delivery efficiency and minimize therapeutic side effects, a variety of delivery vehicles have been developed to encapsulate mRNA, using natural or synthetic materials (e.g. lipids, polymers, etc.) to make nanoparticles with different geometrical structures (nanoparticles, particles, conjugates, etc.) to safely deliver mRNA to target cells to express normal proteins. Among these, lipid nanoparticles (LNPs) have shown potential for efficient delivery as the only delivery system in clinical validation. At the same time, however, the ability of LNPs to target organs other than the liver has been an insurmountable challenge for the industry and has created an invisible ceiling in the mRNA drug development market. When systemically delivered, can LNPs selectively target specific organs, or even further target specific cells in those organs? This metric is the ultimate goal that the entire delivery technology is intended to achieve.

## Conclusion

The introduction of COVID-19 vaccines and the emergence of mRNA companies all point to the fact that mRNA technology is destined to become a spark that can start a new fire, and the widening field of therapeutics and the continued disclosure of data on new therapeutics further demonstrate its endless potential. While the two core technologies of mRNA design and delivery systems are critical to mRNA technology companies, AI technology for R&D platforms is now a strong, if not indispensable, barrier for companies (112). We expect more innovative drug companies to bring together cross-industry and cross-discipline strengths into one, and to overcome the obstacles to bringing innovation to China and the world.

mRNA is one of the most clinically promising frontiers of future biopharmaceuticals, and after years of development, heavyweight products have emerged, showing unprecedented applications in the treatment of metabolic diseases and the prevention of infectious diseases (113). The COVID-19 epidemic has brought mRNA vaccines to widespread public attention and brought about the rapid development of mRNA products, which are also becoming a focus of biopharmaceutical investment and a hotspot for pharmaceutical companies’ research and development. We hope that more people will use this review to understand the opportunities and challenges facing mRNA drugs.

## Author contributions

Conceptualization, QD, TH, QZ, XJ, FC, and XC. Investigation, QD, TH, QZ, and XJ. Writing-original draft preparation, QD, FC, and XC. Writing-review and editing, TH, QZ, XJ, and FC. Funding acquisition, XC. All authors have read and agreed to the published version of the manuscript.

## Acknowledgments

This review is thanks to Wu Jieping Medical Foundation Clinical Research Grant(320.6750.19093-13).

## Conflict of interest

The authors declare that the research was conducted in the absence of any commercial or financial relationships that could be construed as a potential conflict of interest.

## Publisher’s note

All claims expressed in this article are solely those of the authors and do not necessarily represent those of their affiliated organizations, or those of the publisher, the editors and the reviewers. Any product that may be evaluated in this article, or claim that may be made by its manufacturer, is not guaranteed or endorsed by the publisher.
